# Apolipoprotein E Deficiency Causes Endothelial Dysfunction in the Mouse Retina

**DOI:** 10.1155/2019/5181429

**Published:** 2019-11-12

**Authors:** Jenia Kouchek Zadeh, Mayagozel B. Zhutdieva, Panagiotis Laspas, Can Yuksel, Aytan Musayeva, Norbert Pfeiffer, Christoph Brochhausen, Matthias Oelze, Andreas Daiber, Ning Xia, Huige Li, Adrian Gericke

**Affiliations:** ^1^Department of Ophthalmology, University Medical Center, Johannes Gutenberg University Mainz, Langenbeckstr. 1, 55131 Mainz, Germany; ^2^Institute of Pathology, University Medical Center, Johannes Gutenberg University Mainz, Langenbeckstr. 1, 55131 Mainz, Germany; ^3^Institute of Pathology, University of Regensburg, Franz-Josef-Strauss-Allee 11, 93053 Regensburg, Germany; ^4^Center of Cardiology 1, Molecular Cardiology, University Medical Center, Johannes Gutenberg University Mainz, Langenbeckstr. 1, 55131 Mainz, Germany; ^5^Department of Pharmacology, University Medical Center, Johannes Gutenberg University Mainz, Obere Zahlbacher Str. 67, 55131 Mainz, Germany

## Abstract

**Objective:**

Atherogenic lipoproteins may impair vascular reactivity consecutively causing tissue damage in multiple organs via abnormal perfusion and excessive reactive oxygen species generation. We tested the hypothesis that chronic hypercholesterolemia causes endothelial dysfunction and cell loss in the retina.

**Methods:**

Twelve-month-old apolipoprotein E-deficient (ApoE-/-) mice and age-matched wild-type controls were used in this study (*n* = 8 per genotype for each experiment). Intraocular pressure, blood pressure, and ocular perfusion pressure were determined. Retinal arteriole responses were studied *in vitro*, and reactive oxygen and nitrogen species were quantified in the retinal and optic nerve cryosections. The expression of the lectin-like oxidized low-density lipoprotein receptor-1 (LOX-1) and the NADPH oxidase isoforms, NOX1, NOX2, and NOX4, were determined in retinal cryosections by immunofluorescence microscopy. Pro- and antioxidant redox genes were quantified in retinal explants by PCR. Moreover, cell number in the retinal ganglion cell layer and axon number in the optic nerve was calculated.

**Results:**

Responses to the endothelium-dependent vasodilator, acetylcholine, were markedly impaired in retinal arterioles of ApoE-/- mice (*P* < 0.01). LOX-1 (*P* = 0.0007) and NOX2 (*P* = 0.0027) expressions as well as levels of reactive oxygen species (*P* = 0.0022) were increased in blood vessels but not in other retinal structures. In contrast, reactive nitrogen species were barely detectable in both mouse genotypes. Messenger RNA for HIF-1*α*, VEGF-A, NOX1, and NOX2, but also for various antioxidant redox genes was elevated in the retina of ApoE-/- mice. Total cell number in the retinal ganglion cell layer did not differ between ApoE-/- and wild-type mice (*P* = 0.2171). Also, axon number in the optic nerve did not differ between ApoE-/- and wild-type mice (*P* = 0.6435).

**Conclusion:**

Apolipoprotein E deficiency induces oxidative stress and endothelial dysfunction in retinal arterioles, which may trigger hypoxia in the retinal tissue. Oxidative stress in nonvascular retinal tissue appears to be prevented by the upregulation of antioxidant redox enzymes, resulting in neuron preservation.

## 1. Introduction

Hypercholesterolemia is a main risk factor for atherosclerosis and thus a primary cause of cardiovascular organ dysfunction [1–3]. Critical molecular events in atherogenesis are oxidative alterations of lipoproteins and phospholipids, activation of endothelial cells, and infiltration of the vascular wall by macrophages, which is facilitated by reactive oxygen species (ROS) [4, 5]. In the human retina, elevated serum cholesterol levels have been associated with reduced retinal vascular hyperemic responses to flicker light stimulation [6, 7]. Also, familial risk for cardiovascular disease was reported to be associated with alterations in the retinal vascular function [8]. In addition, hypercholesterolemia has been linked to the pathogenesis of retinal artery and vein occlusion, which constitute major reasons for severe visual impairment and blindness [9–11]. Moreover, a recent meta-analysis reported on an association between hyperlipidemia and an increased risk of glaucoma [12], which is one of the leading causes of vision impairment worldwide characterized by progressive loss of retinal ganglion cells (RGCs), visual field defects, and specific morphological changes of the optic nerve [13–15]. One of the heavily discussed risk factors for glaucoma is impaired ocular perfusion, and vascular endothelial dysfunction is suggested to contribute to abnormalities in ocular perfusion observed in glaucoma patients [16, 17]. Hence, hypercholesterolemia might be linked to glaucoma via inducing vascular endothelial dysfunction in the eye. Despite these findings, the specific effects of hypercholesterolemia on retinal vascular function are unknown at the molecular level. Moreover, it remains to be established whether chronic hypercholesterolemia has an influence on RGC viability. Hence, the aim of the present study was to test the hypothesis that chronic hypercholesterolemia affects retinal arteriole reactivity and RGC survival. We used apolipoprotein E-deficient mice (ApoE-/-) for our studies, because they develop spontaneous severe hypercholesterolemia and atherosclerotic lesions in various blood vessels similar to those found in humans [18–20].

## 2. Materials and Methods

### 2.1. Animals

All animals were treated in accordance with the guidelines of EU Directive 2010/63/EU for animal experiments and were approved by the Animal Care Committee of Rhineland-Palatinate, Germany. Mice deficient in the gene coding for apolipoprotein E (ApoE-/-) and age-matched wild-type controls (C57BL/6J) were obtained from The Jackson Laboratory, Bar Harbour, ME, USA. Male mice were fed with a standard rodent chow (Altromin, Lage, Germany) and used for experiments at the age of 12 months. In a previous study using mice from our mouse stock and the same chow, plasma low-density lipoprotein (LDL) and total cholesterol levels were increased by more than 5-fold in 6-month-old ApoE-/- mice compared to wild-type controls [21]. Mice were housed under standardized conditions (12 hours light/dark cycle, temperature of 22 ± 2°C, humidity of 55 ± 10%, and free access to food and tap water).

### 2.2. Measurement of Intraocular Pressure, Blood Pressure, and Cholesterol

Intraocular pressure (IOP) was measured noninvasively in conscious mice (*n* = 8 per genotype) using the Icare® TONOLAB rebound tonometer (Bon Optic, Lübeck, Germany) designed for mice and rats. Before each examination, topical anaesthesia (proparacaine 0.5% eye drops, URSAPHARM Arzneimittel GmbH, Saarbrücken, Germany) was applied onto the ocular surface. Per eye, 12 IOP values were taken and the overall mean of all 24 measurements was calculated for each mouse. Blood pressure measurements with a computerized tail-cuff system (CODA® Monitor, Kent Scientific, Torrington, CT, USA) were conducted in conscious restrained mice (*n* = 8 per genotype). Before measurement, mice were trained for two consecutive days to become acclimatized to the procedure. Mice were placed in restraint tubes to prevent excessive movement during measurement and placed on a warming platform (32-35°C). After tails were cuffed, an acclimatization time of 5 minutes allowed mice to warm up before the start of the experiment. Each session consisted of 20 measuring cycles, of which the first 5 cycles were used for acclimatization and were excluded from the analysis. The average of the following 15 cycles was taken as the reading for each mouse. Ocular perfusion pressure was expressed as the difference between arterial blood pressure and intraocular pressure (IOP). We calculated systolic, diastolic, and mean ocular perfusion pressure, respectively. After mice (*n* = 8 per genotype) had been killed by CO_2_ exposure, blood was collected from the heart, and serum total cholesterol was determined using the scil Reflovet® Plus (scil animal care company GmbH, Viernheim, Germany).

### 2.3. Measurements of Retinal Arteriole Reactivity

Retinal arteriole reactivity was measured in isolated retinas using videomicroscopy as previously described [22, 23]. First, mice (*n* = 8 per genotype) were sacrificed by CO_2_ exposure, and per mouse one eye was isolated and put into cold Krebs–Henseleit buffer. After preparation of the ophthalmic artery, isolation of the retina, cannulation of the ophthalmic artery, and placing the retina onto a transparent plastic platform, retinal arterioles were pressurized to 50 mm Hg. First-order retinal arterioles were then imaged under bright-field conditions and their responses measured after 30 minutes of equilibration. Concentration-response curves for the thromboxane mimetic, U46619 (10^−11^ to 10^−6^ M; Cayman Chemical, Ann Arbor, MI, USA), were conducted. Vessels were then preconstricted to 50–70% of the initial luminal diameter by titration of U46619 and responses to the endothelium-dependent vasodilator, acetylcholine (10^−9^ to 10^−4^ M; Sigma-Aldrich, Taufkirchen, Germany), and to the endothelium-independent nitric oxide (NO) donor, sodium nitroprusside (SNP, 10^−9^ to 10^−4^ M, Sigma-Aldrich), were measured.

### 2.4. Quantification of Reactive Oxygen Species

ROS formation was determined in 10 *μ*m cryosections of the retina and optic nerve by dihydroethidium- (DHE-, 1 *μ*m) derived fluorescence according to a modified protocol for vascular cryosections (*n* = 8 per genotype) [24]. In retinal sections, the fluorescence (518 nm/605 nm excitation/emission) was measured in blood vessels and in individual layers of the inner retina as previously described [25–27].

### 2.5. Immunfluorescence Analysis

Immunostainings were used to quantify reactive nitrogen species (RNS) in cryopreserved retinal and optic nerve cross-sections (*n* = 8 per genotype) stained with an antibody directed against nitrotyrosine (for details see Table 1). Moreover, antibodies directed against the isoforms of nicotinamide adenine dinucleotide phosphate oxidase (NOX), NOX1, NOX2, and NOX4, respectively, and against the lectin-like oxidized low-density lipoprotein receptor-1 (LOX-1) were used to quantify the respective proteins in retinal cross-sections of 7 *μ*m thickness (for antibody details see Table 1). Fixation of tissue sections for 20 minutes in paraformaldehyde (4%) was followed by preincubation with bovine serum albumin (1%) for 30 minutes and then by the respective primary antibody for 2 hours at room temperature. All primary antibodies displayed pronounced immunoreactivity in the thoracic aorta from ApoE-/- mice (positive control), but not from wild-type mice, at the concentrations used for retinal immunostainings, suggesting that they were suitable to detect the respective gene products. After washing the slides in PBS (3 × 5 min), the secondary antibody was applied for 1 hour at room temperature (for details see Table 1). Negative control sections were incubated with a blocking medium and the secondary antibody. Finally, slides were washed in PBS (3 × 5 min) and were mounted using VECTASHIELD® Mounting Medium with DAPI (BIOZOL Diagnostica Vertrieb GmbH, Eching, Germany) and cover-slipped. Subsequently, the fluorescence was measured in blood vessels and in individual layers of the inner retina.

### 2.6. Real-Time PCR

Messenger RNA for the hypoxic markers, *HIF-1α* and *VEGF-A*; the prooxidant redox enzymes, *NOX1*, *NOX2*, and *NOX*; the antioxidant redox enzymes, *catalase*, *GPx1*, *HO-1*, *SOD1*, *SOD2* and *SOD3*; and for the nitric oxide synthase (*NOS*) isoforms, *eNOS*, *iNOS*, and *nNOS*, was quantified in the retina of ApoE-/- and wild-type mice (*n* = 8 per genotype) by real-time PCR. After mice had died by CO_2_ exposure, the one eye per mouse was immediately excised and transferred into cooled phosphate-buffered solution (PBS, Invitrogen, Karlsruhe, Germany) to isolate the retina under a dissecting microscope. Next, the isolated retina was transferred into 1.5 ml plastic tube, rapidly frozen in liquid nitrogen, and stored at -80°C. Later, tissue samples were homogenized (FastPrep; MP Biomedicals, Illkirch, France), and the expression of genes was measured by SYBR Green-based quantitative real-time PCR, as previously described [28]. RNA was isolated using peqGOLD TriFast™ (PEQLAB) and cDNA was generated with the High-Capacity cDNA Reverse Transcription Kit (Applied Biosystems, Darmstadt, Germany). Real-time PCR reactions were performed on a StepOnePlus™ Real-Time PCR System (Applied Biosystems) using SYBR® Green JumpStart™ Taq ReadyMix™ (Sigma-Aldrich) and 20 ng cDNA. Relative mRNA levels of target genes were quantified using comparative threshold (CT) normalized to housekeeping gene TATA-binding protein (TBP). Messenger RNA expression is presented as the fold-change in ApoE-/- mice versus wild-type mice. The PCR primer sequences are listed in Table 2.

### 2.7. Cell Counting in Retinal Wholemounts

Post mortem, one eye per mouse (*n* = 8 per genotype) was excised and fixed in 4% paraformaldehyde (Sigma-Aldrich) for 30 minutes. Next, a retinal wholemount was prepared in cold PBS, transferred onto a glass slide, and stained with cresyl blue as previously described [29]. After the staining procedure, sixteen predefined areas per wholemount, eight central and eight peripheral, of 150 *μ*m × 200 *μ*m were photographed by a blinded investigator as reported in detail previously [25, 30, 31]. Per photograph, all cresyl blue-positive cells were counted manually by a blinded investigator using the cell counter plugin for ImageJ software (NIH, http://rsb.info.nih.gov/ij/) as previously described [25, 31]. The mean cell density (cells/mm^2^) was calculated and multiplied with the wholemounts' surface area to obtain the total number of cells per retina.

### 2.8. Axon Counting in Optic Nerve Cross-Sections

Per mouse, one optic nerve was isolated (*n* = 8 per genotype), placed in fixative solution, and embedded in agar 100 resin. Afterwards, semithin cross-sections were cut with an ultramicrotome (Ultracut E, Leica, Bensheim, Germany), placed on glass slides, and stained with 1% toluidine blue in 1% sodium borate according to standard protocols. Each cross-section was examined using bright-field microscopy by a blinded investigator. Five nonoverlapping fields of 60 *μ*m × 80 *μ*m (one central and four peripheral) were photographed per cross-section. Axons were counted manually by a blinded investigator using ImageJ software. The mean axon density (axons/mm^2^) was calculated and multiplied by the cross-sectional area to obtain the total number of axons per optic nerve as described recently in detail [25, 31].

### 2.9. Statistical Analysis

Data are presented as mean ± SE, and *n* represents the number of mice per group. Constriction responses to U46619 are presented as percent change in luminal diameter from resting diameter, while responses to SNP and acetylcholine are presented as percent change in luminal diameter from the preconstricted diameter. Comparison between concentration-responses was made using two-way ANOVA for repeated measurements. For comparisons of IOP, blood pressure, ocular perfusion pressure, total serum cholesterol, fluorescent intensity, mRNA expression levels, and cell and axon numbers, an unpaired *t*-test was used. The level of significance was set at 0.05.

## 3. Results

### 3.1. Intraocular Pressure, Blood Pressure, Ocular Perfusion Pressure, and Total Serum Cholesterol

No differences in intraocular pressure, blood pressure, and ocular perfusion pressure were detected between ApoE-/- and wild-type mice. Total serum cholesterol was markedly elevated in ApoE-/- mice compared to wild-type mice (*P* < 0.0001, *n* = 8 per genotype). The data are presented in Table 3.

### 3.2. Responses of Retinal Arterioles

**Figure 1:**
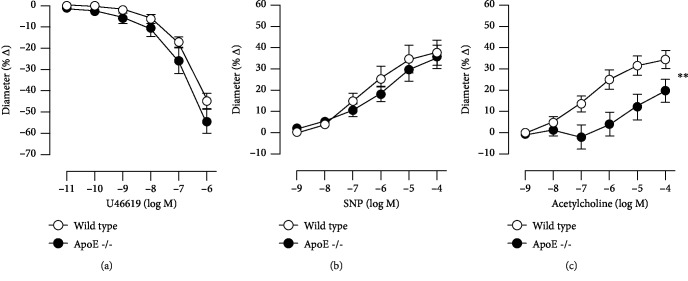
Responses of retinal arterioles from ApoE−/− and wild-type mice to the thromboxane mimetic, U46619 (a), the endothelium-independent vasodilator, SNP (b), and to the endothelium-dependent vasodilator, acetylcholine (c). Values are presented as mean ± SE (*n* = 8 per concentration and genotype; ^∗∗^*P* < 0.01).

The initial luminal diameter of retinal arterioles was similar in both mouse genotypes (20.52 ± 0.9656 *μ*m and 21.58 ± 0.9808 *μ*m in ApoE-/- and wild-type mice, respectively, *P* = 0.4550, *n* = 8 per genotype). U46619 elicited concentration-dependent vasoconstriction of retinal arterioles that was similar in ApoE-/- and wild-type mice (54.50 ± 5.441% versus 44.86 ± 3.495%, ApoE-/- versus wild-type mice at 10^−6^ M; *P* > 0.05; Figure 1(a)). The endothelium-independent vasodilator, SNP, elicited concentration-dependent vasodilation that did also not differ between both mouse genotypes (35.77 ± 5.531% versus 37.70 ± 5.837%, ApoE-/- versus wild-type mice at 10^−4^ M; *P* > 0.05; Figure 1(b)). In contrast, the endothelium-dependent vasodilator, acetylcholine, produced concentration-dependent vasodilation, which was impaired in arterioles from ApoE-/- mice (19.79 ± 5.576% versus 34.41 ± 4.175%, ApoE-/- versus wild-type mice at 10^−4^ M; *P* < 0.01; Figure 1(c)).

### 3.3. ROS and RNS Formation in the Retina and Optic Nerve

**Figure 2:**
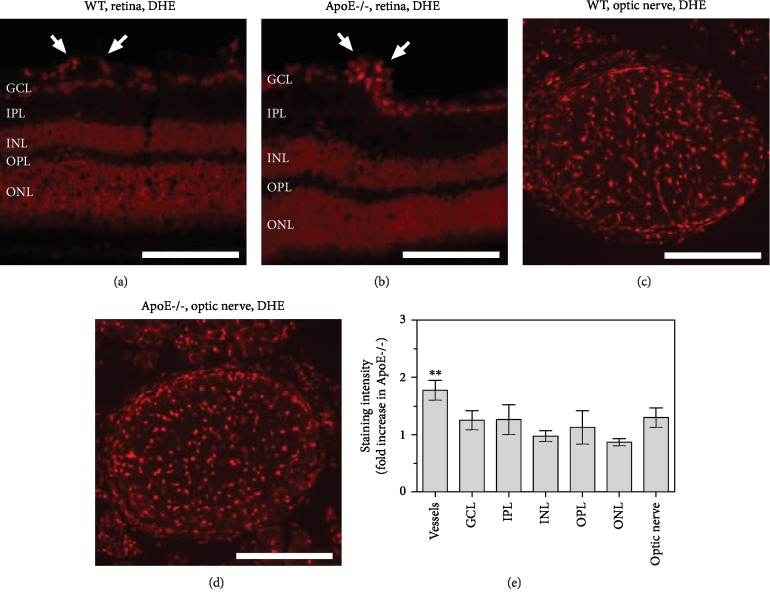
DHE stainings of retinal cross-sections (a, b) and of optic nerve cross-sections (c, d) from wild-type and ApoE-/- mice, respectively. Staining intensity was increased in retinal blood vessels from ApoE-/- mice but did neither differ in any of the retinal layers nor in the optic nerve between both genotypes (e). The white arrows point to retinal blood vessel cross-sections. GCL: ganglion cell layer; IPL: inner plexiform layer; INL: inner nuclear layer; OPL: outer plexiform layer; ONL: outer nuclear layer. Values are presented as mean ± SE (*n* = 8 per genotype; ^∗∗^*P* < 0.01). Scale bar = 100 *μ*m.

**Figure 3:**
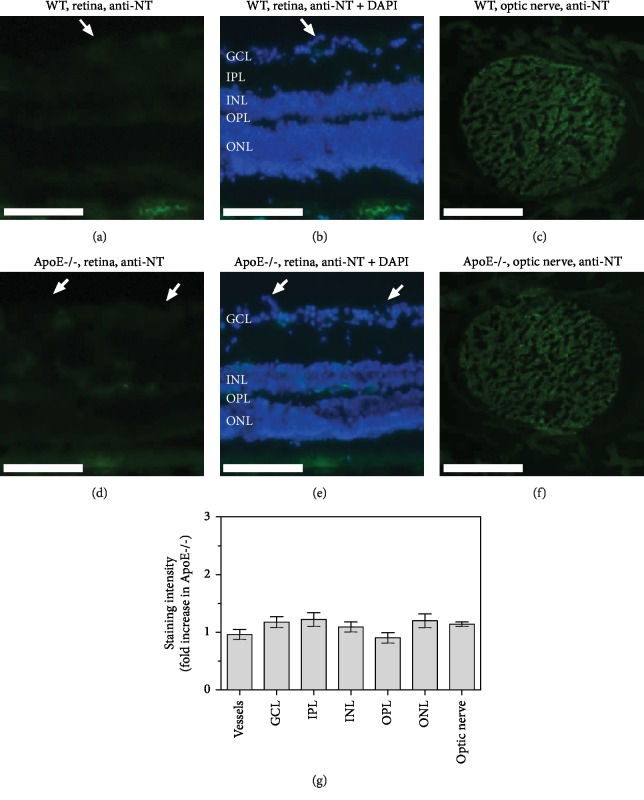
Nitrotyrosine immunostainings (anti-NT) in retinal cross-sections and optic nerve cross-sections from wild-type (a–c) and ApoE-/- mice (d–f). Immunoreactivity to nitrotyrosine was negligible in the inner retina of both genotypes (a, d). In optic nerve cross-sections, some green hyperfluorescent spots were visible, but no differences in fluorescent intensity were observed between both genotypes (g). The white arrows point to retinal blood vessels cross-sections. GCL: ganglion cell layer; IPL: inner plexiform layer; INL: inner nuclear layer; OPL: outer plexiform layer; ONL: outer nuclear layer. Values are presented as mean ± SE (*n* = 8 per genotype). Scale bar = 100 *μ*m.

Staining of retinal sections with DHE revealed increased fluorescence intensity specifically in retinal blood vessels from ApoE−/− mice, indicative of elevated ROS levels (*P* = 0.0022, ApoE-/- versus wild-type mice, *n* = 8 per genotype; Figures 2(a), 2(b), and 2(e)). No differences in fluorescence intensity where found between both genotypes in the individual layers of the inner retina (*n* = 8 per genotype; Figures 2(a), 2(b), and 2(e)). Similarly, in cross-sections of the optic nerve, no differences in DHE fluorescence intensity were found between wild-type and ApoE−/− mice (*n* = 8 per genotype; Figures 2(c)–2(e)). Immunoreactivity to nitrotyrosine was negligible in blood vessels and in all inner retinal layers of both mouse genotypes (*n* = 8 per genotype; Figures 3(a), 3(b), 3(d) and 3(e)), suggesting that RNS levels were very low and not increased in ApoE-/- mice (Figure 3(g)). In optic nerve cross-sections of both genotypes, some green hyperfluorescent spots were visible (Figures 3(c) and 3(f)), but no differences in immunoreactivity to nitrotyrosine were detectable between wild-type and ApoE−/− mice (*n* = 8 per genotype; Figure 3(g)).

### 3.4. NOX1, NOX2, and NOX4 Expressions in the Retina

**Figure 4:**
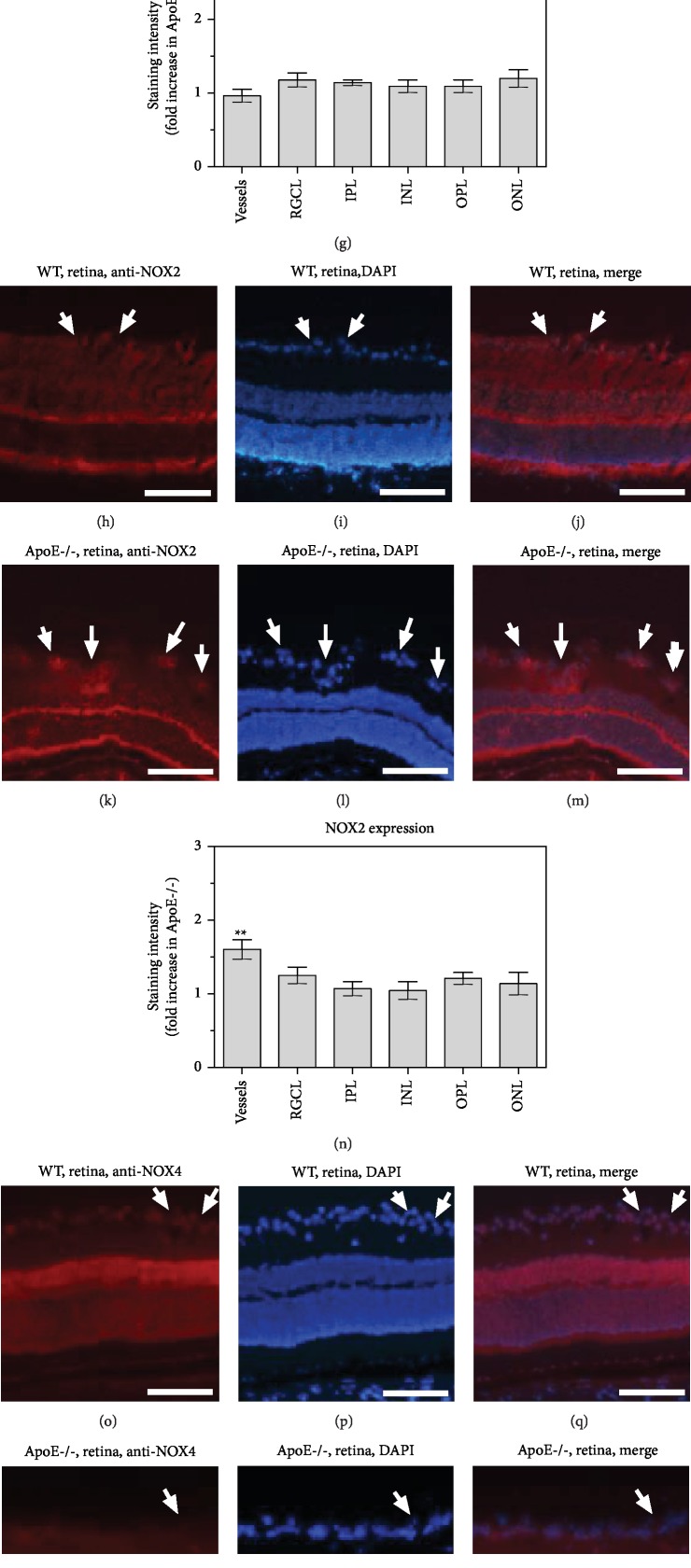
Immunostainings for NOX1 (a–g), NOX2 (h–n), and NOX4 (o–u) in retinal cross-sections from wild-type and ApoE-/- mice. Immunoreactivity to NOX1 was similar in blood vessels and all retinal layers in both mouse genotypes (g). In contrast, immunoreactivity to NOX2 was increased in retinal blood vessels from ApoE-/- mice but did not differ in individual retinal layers of both mouse groups (n). Immunoreactivity to NOX4 was also similar throughout the retina in both genotypes (u). The white arrows point to retinal blood vessel cross-sections. GCL: ganglion cell layer; IPL: inner plexiform layer; INL: inner nuclear layer; OPL: outer plexiform layer; ONL: outer nuclear layer. Values are presented as mean ± SE (*n* = 8 per genotype; ^∗∗^*P* < 0.01). Scale bar = 100 *μ*m.

Immunoreactivity to NOX1 did not differ between blood vessels and any of the inner retinal layers of ApoE−/− and wild-type mice (*n* = 8 per genotype; Figures 4(a)–4(g)). In contrast, immunoreactivity to NOX2 was markedly increased in retinal blood vessels from ApoE-/- mice (*P* = 0.0027, ApoE-/- versus wild-type mice, *n* = 8 per genotype), but did not differ in individual retinal layers of both mouse genotypes (Figures 4(h)–4(n)). NOX4 immunoreactivity was similar in blood vessels and all retinal layers of both mouse genotypes (*n* = 8 per genotype; Figures 4(o)–4(u)).

### 3.5. Expression of LOX-1 in the Retina

**Figure 5:**
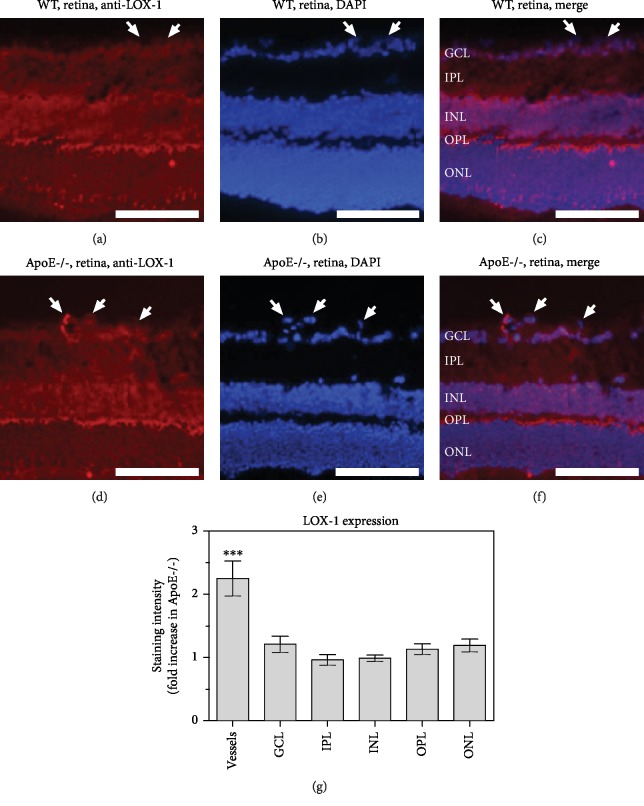
Immunostainings for the ox-LDL receptor, LOX-1, in retinal cross-sections from wild-type (a–c) and ApoE-/- mice (d–f), respectively. Staining intensity was increased in blood vessels from ApoE-/- mice (g) but did not differ in any of the retinal layers between both genotypes. GCL: ganglion cell layer; IPL: inner plexiform layer; INL: inner nuclear layer; OPL: outer plexiform layer; ONL: outer nuclear layer. Values are presented as mean ± SE (*n* = 8 per genotype; ^∗∗∗^*P* < 0.001). Scale bar = 100 *μ*m.

Immunoreactivity to LOX-1, which serves as a receptor for ox-LDL, was faint in the inner retina of wild-type mice. Also, immunoreactivity in blood vessels was not pronounced in retinas of wild-type mice (*n* = 8 per genotype; Figures 5(a)–5(c)). The immunoreactivity pattern in individual retinal layers from ApoE-/- mice resembled the one from wild-type mice. However, strong immunoreactivity was seen in retinal blood vessels from ApoE-/- mice (*n* = 8 per genotype; Figures 5(c)–5(e)). The staining intensity was markedly stronger in retinal blood vessels from ApoE-/- mice compared to wild-type mice (*P* = 0.0007, *n* = 8 per genotype; Figure 5(f)).

### 3.6. Expression of Hypoxic and Redox Genes in the Retina

**Figure 6:**
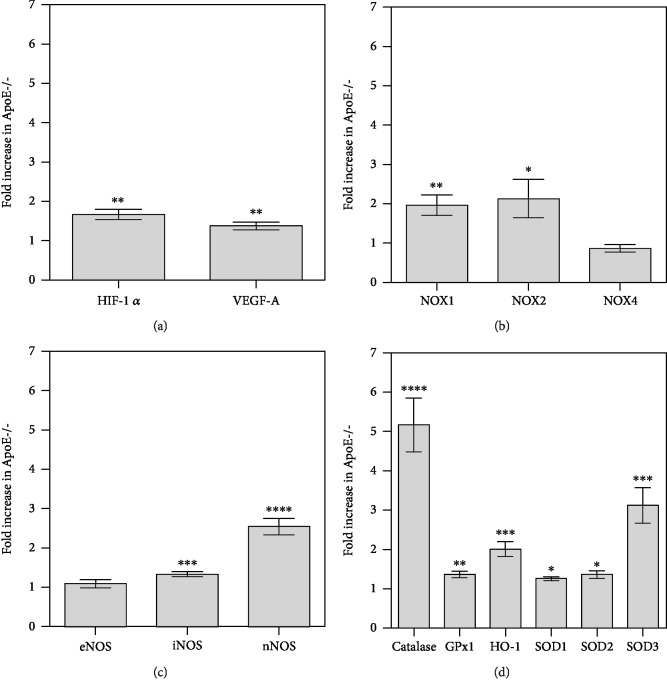
Messenger RNA expression of hypoxic markers ((a) HIF-1*α*, VEGF-A), prooxidant ((b) NOX1, NOX2, and NOX4), the three nitric oxide synthase isoforms ((c) eNOS, iNOS, and nNOS), and of the antioxidant redox enzymes ((d) catalase, GPx1, HO-1, SOD1, SOD2, and SOD3) in retinal samples from wild-type and ApoE-/- mice. Data are presented as the fold-change (mean ± SE) in ApoE-/- versus wild-type mice (*n* = 8 per genotype, ^∗^*P* < 0.05, ^∗∗^*P* < 0.01, ^∗∗∗^*P* < 0.001, ^∗∗∗∗^*P* < 0.0001).

In the retina of ApoE-/- mice, mRNA for both hypoxic markers, *HIF-1α* and *VEGF-A*, was slightly but significantly elevated compared to wild-type mice (1.7-fold for *HIF-1α*, *P* = 0.0013 and 1.4-fold for *VEGF-A*, *P* = 0.0095, *n* = 8 per genotype), indicative of a hypoxic condition (Figure 6(a)). Also, mRNA for the prooxidant redox genes, *NOX1* and *NOX2*, was increased in the retina from ApoE-/- mice compared to wild-type mice (2.0-fold for *NOX1*, *P* = 0.0053 and 2.1-fold for *NOX2*, *P* = 0.0312, *n* = 8 per genotype; Figure 6(b)). Among the three nitric oxide synthase (NOS) isoforms, mRNA expression for inducible (*iNOS*) and neuronal NOS (*nNOS*) was found to be increased (1.3-fold for *iNOS*, *P* = 0.0009 and 2.6-fold for *nNOS*, *P* = <0.0001, *n* = 8 per genotype; Figure 6(c)). Interestingly, also mRNA expression for all antioxidant redox genes tested was elevated (5.2-fold for *catalase*, *P* < 0.0001; 1.4-fold for *GPx-1*, *P* = 0.0063; 2.0-fold for *HO-1*, *P* = 0.0005; 1.3-fold for *SOD1*, *P* = 0.0180; 1.4-fold for *SOD2*, *P* = 0.0383; 3.1-fold for *SOD3*, *P* = 0.0006, *n* = 8 per genotype; Figure 6(d)).

### 3.7. Retinal Ganglion Cell Layer Cells and Optic Nerve Axons

**Figure 7:**
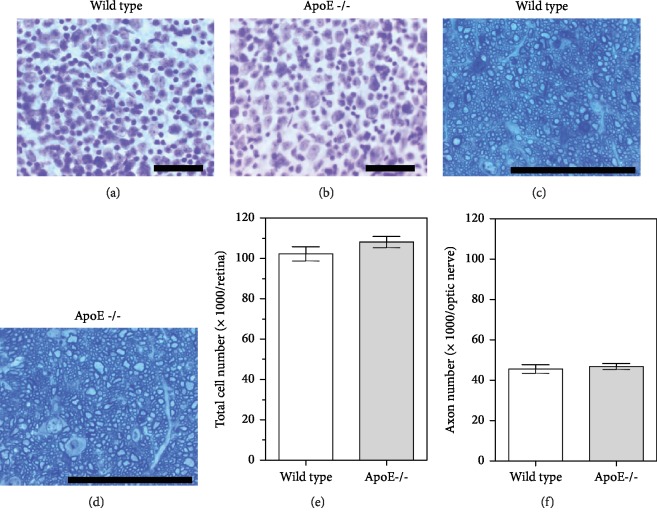
Example photographs taken from retinal wholemounts stained with cresyl blue and optic nerve cross-sections stained with toluidine blue of wild-type (a, c) and ApoE-/- mice (b, d). Total cell number in the RGC layer (e) and axon number (f) in the optic nerve was similar in wild-type and ApoE-/- mice. Values are presented as mean ± SE (*n* = 8 per genotype). Scale bar = 50 *μ*m.

Total cell number in the RGC layer was 108 063 ± 2 745 and 102 255 ± 3 558 in ApoE-/- and wild-type mice and did not differ between both genotypes (*P* = 0.2171, *n* = 8 per genotype; Figure 7). The number of axons in the optic nerve, representing the axons of RGCs, did also not differ between both genotypes. The axon number was 46 790 ± 1 493 and 45 554 ± 2 145 in ApoE-/- and wild-type mice, respectively, and was not different between both mouse genotypes (*P* = 0.6435, *n* = 8 per genotype; Figure 7).

## 4. Discussion

There are several major new findings emerging from this experimental study. First, the lack of apolipoprotein E had no effect on intraocular pressure, blood pressure, and ocular perfusion pressure but affected reactivity of retinal arterioles to the endothelium-dependent vasodilator, acetylcholine, indicative of endothelial dysfunction. Second, ROS levels, but not RNS levels, were found to be elevated in retinal arterioles of ApoE-/- mice. In contrast, neither ROS nor RNS were increased in individual retinal layers and the optic nerve of ApoE-/- mice suggesting that oxidative stress is limited to the vasculature and nitrosative stress is negligible. Also, immunoreactivity to LOX-1 and NOX2, but not to NOX1 or NOX4 was elevated in retinal vessels of ApoE-/- mice, suggesting that a mechanism involving LOX-1, NOX2, and ROS may be involved in mediating endothelial dysfunction. The lack of apolipoprotein E was associated with increased retinal mRNA expression for the hypoxia markers, *HIF-1α* and *VEGF-A*, as well as of redox genes coding for the prooxidant enzymes *NOX1* and *NOX2*. However, mRNA expression for the antioxidant redox genes *SOD1*, *SOD3*, *SOD3*, *GPx1*, *HO-1*, and *catalase* were also increased, suggesting that enhanced ROS production is associated with a compensation by antioxidant enzymes. Third, total cell number in the RGC layer and axon number in the optic nerve was not affected by the lack of apolipoprotein E. These findings illustrate that apolipoprotein E deficiency causes oxidative stress and endothelial dysfunction in retinal arterioles, but no oxidative damage in nonvascular retinal tissue probably by effective buffering of excessive ROS and RNS generation by antioxidant redox enzymes.

During hypercholesterolemia, oxidized low-density lipoproteins (ox-LDLs) have been shown to trigger the expression of prooxidant enzymes and thus, ROS generation, in the vascular wall via involvement of LOX-1 [32]. Of note, the expression LOX-1, which serves as a receptor for ox-LDL, was reported to be upregulated in hypercholesterolemia via positive feedback mechanisms involving the transcription factor NF-*κ*B [33, 34]. In agreement with these studies, we found increased LOX-1 expression and increased ROS levels in the vascular wall of retinal blood vessels from ApoE-/- mice. In many vascular beds, high ROS concentrations elicit endothelial dysfunction, reflected by a reduced endothelium-dependent vasodilation, in part by affecting eNOS bioactivity and by inactivation of nitric oxide [32, 35, 36]. These mechanisms have also been described in cerebral blood vessels of various hypercholesterolemic animal models, including ApoE-/- mice [37–40]. Likewise, in the human retina, elevated serum cholesterol levels have been associated with reduced retinal vascular hyperemic responses to flicker light stimulation, which are in part nitric oxide synthase-dependent [6, 7]. However, the molecular effects of hypercholesterolemia on retinal endothelial function have not been elucidated so far. The findings of the present study suggest that LOX-1, NOX2, and ROS are involved in mediating hypercholesterolemia-induced endothelial dysfunction in the retina, which is in concert with a study in cerebral blood vessels reporting that NOX2-derived ROS abrogated nitric oxide function in ApoE-/- mice [40].

We excluded the possibility that endothelial dysfunction was triggered by differences in IOP, blood pressure, or ocular perfusion pressure between ApoE-/- and wild-type mice. Arterial hypertension is a trigger factor of endothelial dysfunction in various blood vessels, including ocular and cerebral vessels [41, 42]. Conversely, low ocular perfusion pressure was associated with glaucoma prevalence, incidence, and progression [43]. Similar to the present findings, most of the previous studies reported that blood pressure does not differ in ApoE-/- and wild-type mice and is stable with age [20, 44–46]. Interestingly, other previous studies in ApoE-/- mice revealed that some small blood vessels do not develop endothelial dysfunction [47, 48]. One possible explanation for these findings is that endothelium-derived hyperpolarizing factor- (EDHF-) dependent vasodilation mechanisms are less affected by hypercholesterolemia [47]. In mouse retinal arterioles, however, endothelium-dependent vasodilation is mainly mediated by eNOS and, during eNOS deficiency, by nNOS and COX-2 metabolites, suggesting that EDHF pathways play only a negligible role [49, 50].

An intriguing question concerning many retinal diseases is how endothelial dysfunction affects neuron survival. So far only indirect links between impaired vascular responses and the onset and progression retinal pathologies exist. For example, reduced responses of retinal arterioles to various stimuli have been reported in patients with diabetic retinopathy and glaucoma [51, 52]. Other studies in humans suggest that certain polymorphisms of the gene coding for eNOS, which plays a major role in endothelial function of retinal arterioles, have a risk association for onset or progression of diabetic retinopathy and of some forms of glaucoma [53–57]. However, in animal models characterized by reduced responsiveness to the endothelium-dependent vasodilator, acetylcholine, such as eNOS-deficient and M_3_ receptor-deficient mice, no loss of RGCs has been detected [22, 25, 30, 50]. On the other hand, in diabetic mice, eNOS deficiency was reported to promote the progression of diabetic retinopathy, suggesting that endothelial dysfunction might accelerate pathophysiological processes in the retina [58].

The retinal vasculature supplies the inner retinal layers, while the outer layers are supplied by choroidal blood vessels [59]. Hence, impaired blood supply due to abnormal reactivity of retinal vessels is supposed to affect primarily the inner retinal cell layers, such as the RGC layer. Intriguingly, previous studies in genetically modified animal models of atherosclerosis, including ApoE-/- mice, reported on pathological changes in outer retinal layers, such as lipoidal degenerations and basal deposits in the Bruch's membrane that resemble alterations observed in ageing human eyes, with some functional and morphologic alterations similar to those found in age-related macular degeneration [60–63]. In support of this concept, some studies in humans found a positive association of serum cholesterol levels with age-related macular degeneration [64, 65].

In contrast, the effects of apolipoprotein E deficiency on RGC survival have not been studied in detail so far. A recent meta-analysis reported that hyperlipidemia was associated with an increased risk of glaucoma, a disease characterized by progressive RGC and visual field loss [12]. However, the original studies included in the meta-analysis displayed highly heterogenic results [12]. Also, studies on the association of apolipoprotein E gene polymorphisms with glaucoma reported heterogenic results [66–69]. Interestingly, a study in mice found that apolipoprotein E deficiency was even protective against RGC death induced by elevated intraocular pressure or optic nerve crush [70]. In the present study, 12-month-old ApoE-/- mice and age-matched wild-type controls had a similar total cell number in the RGC layer, which comprises RGCs, displaced amacrine cells, vascular cells, and glial cells. Moreover, no differences in optic nerve axon number, which reflects the number of RGCs, have been detected, suggesting that apolipoprotein E deficiency has no effect of RGC viability. The mRNA expression data of the present study, however, revealed increased expression levels for *HIF-1α* and *VEGF-A* together with increased levels for *NOX1* and *NOX2* indicating that abnormal vascular function in the retina may have triggered hypoxia and ROS generation. However, also a variety of antioxidant redox genes was shown to be upregulated in the retina of ApoE-/- mice, suggesting that compensatory antioxidant pathways have been activated, which may have buffered excessive ROS and RNS generation and, thus, their potential deleterious effects on cell viability.

Apart from its role in the regulation of cholesterol homeostasis in the peripheral circulation, apolipoprotein E is expressed in the central nervous system including the retina and optic nerve, where it takes part in cholesterol transport and intracellular exchange of metabolites between neurons and glial cells [71–73]. One study reported that activated retinal glial cells promote neurite outgrowth in RGCs via involvement of apolipoprotein E [73]. However, studies of retinal histology and function have shown only minor changes in ApoE-/- mice [74, 75]. In support of this concept, detailed neurocognitive and retinal studies in a 40-year-old patient with severe dysbetalipoproteinemia due to total absence of apolipoprotein E failed to demonstrate any functional and morphological defects [76]. These findings suggest that redundant mechanisms exist in the retina to compensate for the lack of apolipoprotein E.

## 5. Conclusions

Our study demonstrates that chronic apolipoprotein E deficiency promotes endothelial dysfunction in retinal arterioles. The presented data also suggest that LOX-1, NOX2, and ROS, but not RNS, are involved in this process. Although the mRNA expression for prooxidant enzymes was increased in the retina of ApoE-/- mice, mRNA for antioxidant enzymes was also upregulated, indicating that oxidative stress in retinal tissue appears to be quenched by antioxidant mechanisms, which results in preservation of RGC viability. Hence, our data also suggest that apolipoprotein E deficiency and endothelial dysfunction of the retinal vasculature are not deleterious to RGCs, at least in the absence of additional pathophysiological stimuli.

## Figures and Tables

**Table 1:** Specifications of antibodies used for immunofluorescence analysis.

Antibody	Article number, company	Species, clonality	Dilution
Nitrotyrosine	06-284, Merck Millipore, Darmstadt, Germany	Rabbit, polyclonal	1 : 100
NOX1	ab131088, Abcam, Waltham, MA, USA	Rabbit, polyclonal	1 : 200
NOX2	ab129068, Abcam, Waltham, MA, USA	Rabbit, monoclonal	1 : 200
NOX4	ab109225, Abcam, Waltham, MA, USA	Rabbit, monoclonal,	1 : 200
LOX-1	bs-2044R, Biozol Diagnostica Vertrieb GmbH, Eching, Germany	Rabbit, polyclonal	1 : 100
FITC-coupled secondary antibody (for nitrotyrosine staining)	111-095-003, dianova GmbH, Hamburg, Germany	Goat anti-rabbit, polyclonal	1 : 200
Rhodamine red-X-coupled secondary antibody (for NOX1, NOX, NOX4 and LOX-1 staining)	111-295-003, dianova GmbH, Hamburg, Germany	Goat anti-rabbit, polyclonal	1 : 200

**Table 2:** Sequences of the primers used for real-time PCR analysis.

Gene	Forward	Reverse
*HIF-1α*	TCATCAGTTGCCACTTCCCCAC	CCGTCATCTGTTAGCACCATCAC
*VEGF-A*	ACTTGTGTTGGGAGGAGGATGTC	AATGGGTTTGTCGTGTTTCTGG
*NOX1*	GGAGGAATTAGGCAAAATGGATT	GCTGCATGACCAGCAATGTT
*NOX2*	CCAACTGGGATAACGAGTTCA	GAGAGTTTCAGCCAAGGCTTC
*NOX4*	TGTAACAGAGGGAAAACAGTTGGA	GTTCCGGTTACTCAAACTATGAAGAGT
*eNOS*	CCTTCCGCTACCAGCCAGA	CAGAGATCTTCACTGCATTGGCTA
*iNOS*	CAGCTGGGCTGTACAAACCTT	CATTGGAAGTGAAGCGTTTCG
*nNOS*	TCCACCTGCCTCGAAACC	TTGTCGCTGTTGCCAAAAAC
*Catalase*	CAAGTACAACGCTGAGAAGCCTAAG	CCCTTCGCAGCCATGTG
*GPx1*	CCCGTGCGCAGGTACAG	GGGACAGCAGGGTTTCTATGTC
*HO-1*	GGTGATGCTGACAGAGGAACAC	TAGCAGGCCTCTGACGAAGTG
*SOD1*	CCAGTGCAGGACCTCATTTTAAT	TCTCCAACATGCCTCTCTTCATC
*SOD2*	CCTGCTCTAATCAGGACCCATT	CGTGCTCCCACACGTCAAT
*SOD3*	TTCTTGTTCTACGGCTTGCTACTG	AGCTGGACTCCCCTGGATTT
*TBP*	CTT CGT GCA AGA AAT GCT GAA T	CAG TTG TCC GTG GCT CTC TTA TT

**Table 3:** Intraocular pressure, blood pressure, ocular perfusion pressure, and total serum cholesterol in wild-type and ApoE-/- mice (*n* = 8 per genotype).

Systemic parameters	Wild type	ApoE-/-	*P* value
Intraocular pressure (mm hg)	11.95 ± 0.5491	11.56 ± 0.6165	0.6428
Blood pressure (mm hg)			
Systolic	98.68 ± 4.341	105.7 ± 4.263	0.2701
Diastolic	67.74 ± 3.959	63.23 ± 4.606	0.4702
Mean	77.73 ± 3.907	77.06 ± 3.941	0.9051
Ocular perfusion pressure (mm hg)			
Systolic	86.73 ± 4.024	94.12 ± 3.925	0.2098
Diastolic	55.79 ± 3.631	51.68 ± 4.390	0.4817
Mean	65.79 ± 3.568	65.50 ± 3.647	0.9566
Total cholesterol (mg/dL)	145.1 ± 5.642	511.0 ± 12.21	**<0.0001**

## Data Availability

The data used to support the findings of this study are available from the corresponding author upon request.

## Abbreviations

ApoE-/-: Apolipoprotein E-deficient mouse
DAPI: 4′,6-Diamidino-2-phenylindole
DHE: Dihydroethidium
EDHF: Endothelium-derived hyperpolarizing factor
eNOS: Endothelial nitric oxide synthase
GPx1: Glutathinone peroxidase 1
HIF-1*α*: Hypoxia-inducible factior-1*α*
HO-1: Heme oxygenase-1
iNOS: Inducible nitric oxide synthase
IOP: Intraocular pressure
nNOS: Neuronal nitric oxide synthase
NOX: Nicotinamide adenine dinucleotide phosphate oxidase
LDL: Low-density lipoprotein
LOX-1: Lectin-like oxidized low-density lipoprotein receptor-1
PBS: Phosphate-buffered saline
RGC: Retinal ganglion cell
RNS: Reactive nitrogen species
ROS: Reactive oxygen species
SNP: Sodium nitroprusside
SOD: Superoxide dismutase
TNF-*α*: Tumor necrosis factor alpha
U46619: 9,11-Dideoxy-9*α*,11*α*-methanoepoxy prostaglandin F2*α*
VEGF-A: Vascular endothelial growth factor a.
